# Field testing of a household-scale onsite blackwater treatment system in South Africa

**DOI:** 10.1016/j.scitotenv.2019.135469

**Published:** 2020-02-10

**Authors:** Tapuwa Sahondo, Sarah Hennessy, Rebecca C. Sindall, Hitendra Chaudhari, Stephanie Teleski, Brendon J. Lynch, Katelyn L. Sellgren, Brian R. Stoner, Sonia Grego, Brian T. Hawkins

**Affiliations:** a Pollution Research Group, University of KwaZulu-Natal, Durban, South Africa; b Triangle Environmental Health Initiative, Durham, NC, USA; c Biomass Controls, PBC, Durham, NC, USA; d Duke University Center for WaSH-AID, Department of Electrical and Computer Engineering, Durham, NC, USA

**Keywords:** WASH, Onsite sanitation, Blackwater reuse, Durban, User testing

## Abstract

Innovations that enable cost-effective and resource-conserving treatment of human waste are required for the 4.2 billion people in the world who currently lack safe and reliable sanitation services. Onsite treatment and reuse of blackwater is one strategy towards this end, greatly reducing the need to transport wastewater over long distances either via sewers or trucks. Here, we report on the field testing of a prototype onsite blackwater treatment system conducted over a period of 8 months. The system was connected to a women's toilet in a public communal ablution block located in an informal settlement near Durban, South Africa. Liquid waste was treated by separation and diversion of large solids, settling of suspended solids, and filtration through activated carbon prior to disinfection by electrochemical oxidation. System performance was monitored daily by measurement of chemical and physical water quality parameters onsite and confirmed by periodic detailed analysis of chemical and biological parameters at an offsite lab. Daily monitoring of system performance indicated that the effluent had minimal color and turbidity (maximum 90 Pt/Co units and 6.48 NTU, respectively), and consistent evolution of chlorine as blackwater passed through the system. Weekly offsite analysis confirmed that the system consistently inactivated pathogens (*E. coli* and coliforms) and reduced chemical oxygen demand and total suspended solids to meet ISO 30500 category B standards. Significant reductions in total nitrogen load were also observed, though these reductions often fell short of the 70% reduction required by ISO 30500. No significant reduction in total phosphorus was observed. Maintenance requirements were identified, and the resilience of the system to restart following a prolonged shutdown was demonstrated, but significant improvements are required in the design of the solid/liquid separation mechanism for application of this system in a wiping culture.

## Highlights

• 4.5 billion people worldwide lack access to safely managed sanitation.
• One approach to improving access is decentralized/onsite wastewater treatment.
• An onsite blackwater treatment system was field tested in Durban, South Africa.
• Disinfection efficacy and improved water quality were demonstrated in the field.
• With further design refinements this system will enable onsite blackwater reuse.

## Graphical abstract

**Figure:**
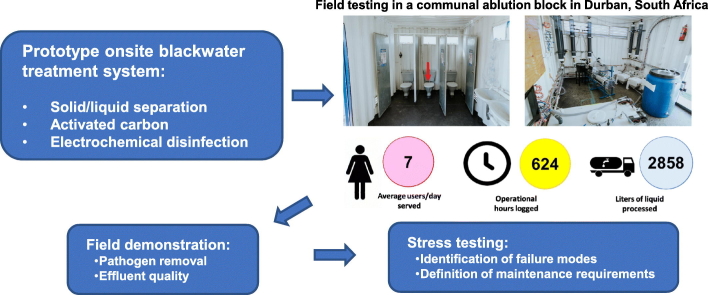

## 1 Introduction

It is presently estimated that 4.2 billion people lack access to safely managed sanitation services, leading to the spread of diarrheal diseases that increase child mortality, stunt growth, and hinder education and economic development (WHO, 2012; UNICEF and WHO, 2019). A large proportion of this global population lives in rapidly growing urban and peri-urban areas, including informal settlements. Informal settlements are defined by the 2001 South African Census as ‘An unplanned settlement on land which has not been surveyed or proclaimed as residential, consisting mainly of informal dwellings (shacks)’ (Housing Development Authority, 2012). The 2009 National Housing Code's Informal Settlement Upgrading Programme uses the following criteria in identifying informal settlements; illegality and informality; inappropriate locations; restricted public and private sector investment; poverty and vulnerability; and social stress. These settlements normally lack adequate municipal services although many settlements in South Africa are provided with basic services such as communal tap points and rudimentary sanitation (Braathen et al., 2016). As a result, sanitation is a major issue for these communities, posing significant public health risks and loss of dignity (Sutherland et al., 2014).

Durban, South Africa, like many African cities, has a growing population due to natural growth and migration to urban areas (eThekwini Municipality, 2019b). Challenges of a fast-growing population include unemployment, poverty and an increase in housing and services backlogs (Sutherland et al., 2014). Informal settlements represent ~1/3 of housing units in eThekwini Municipality—many in steep or flood prone locations or beyond the waterborne edge—and it is estimated that it will take decades to clear this housing backlog (eThekwini Municipality, 2019b).

The Constitution of South Africa (1996) states that water is a right for all. As government policies developed, the responsibility to provide water and sanitation services fell on municipalities. Municipalities act as the water service authorities that are required to plan and implement water and sanitation provision (Sutherland et al., 2014). Though they have access to government subsidies through the Municipal Infrastructure Grant (MIG), municipalities are expected to make up the financial shortfalls (Gounden et al., 2006). With an increasing population with limited capacity to pay for services under their jurisdiction, pressure to reduce costs in water and sanitation services remains high.

In order to meet sanitation demands, South African municipalities have implemented different sanitation technologies. These include community-built pit latrines, urine diverting dry (UDD) toilets, and Communal Ablution Blocks (CABs). Both pit latrines and UDD toilets are dry (non-flush) toilet systems which require expensive and hazardous emptying and offer less desirable user experiences than flush toilets (Duncker et al., 2007; Gounden et al., 2006). CABs are shared facilities in informal settlements with separated male and female ablution blocks. CABs require a connection to municipal water and sewer lines but are designed to serve 50 households within 200 m of the CAB. Each block houses 4 flush toilets, 2 hand-washing basins, 2 showers, 2 laundry sinks and 2 urinals for the male block. CABs are installed under the Interim Services Programme provided under the municipal administration of eThekwini Water and Sanitation (EWS); EWS is responsible for water and sanitation provision across eThekwini Municipality (Sutherland et al., 2014). As such, CABs are regarded as temporary structures to be removed as the government national housing plans are implemented.

In light of the water scarcity experienced in South Africa and the increasing pressure to provide growing informal settlements with their right to adequate sanitation, EWS supported non-conventional sanitation treatment options to be field tested in Durban. Onsite treatment systems provide solutions by offering the preferred flush toilets with less load on centralized municipal water and sanitation plants (Truyens et al., 2018). Onsite sanitation technologies which are affordable, robust, treat waste to municipal standards, require little to no operation and maintenance and do not require user adaptability were welcomed to Durban by EWS for field testing. The successful implementation of these sanitation systems requires not only technical and economic feasibility but also social suitability and acceptance (Ashipala and Armitage, 2011). As interest in these onsite technologies is high, testing systems in the Durban environment allows for rapid transition from a laboratory prototype to a marketable product.

The Duke University Center for Water, Sanitation, Hygiene, and Infectious Disease (WaSH-AID) has developed an onsite treatment system for toilets that separates solids from liquids in the blackwater waste stream. The liquid fraction is disinfected by electrochemical oxidation of the chloride in urine to chlorine (Sellgren et al., 2017), and is designed to re-use the treated liquids onsite as flush water, thus reducing and eventually eliminating the need for piped-in water and a sewer connection or a pit requiring frequent emptying (Hawkins et al., 2017). Addition of activated carbon filters to this system improved the effluent quality by reducing chemical oxygen demand (COD) and total suspended solids (TSS) and increasing the energy efficiency of the electrochemical oxidation process (Rogers et al., 2018). Several options for remediation of the separated solids are under development, including drying for combustion and anaerobic digestion; the solid remediation system development and testing will be described in separate reports.

Here, we report on an eight-month field test of this improved liquid treatment system connected to a toilet in a women's CAB located in an informal settlement near Durban. The aims of this field study were twofold: 1) to evaluate the system's functionality in a less controlled environment than a laboratory, and 2) to identify the maintenance requirements and component life-cycle assessments of the system.

## 2 Materials and methods

### 2.1 Prototype system

**Fig. 1:**
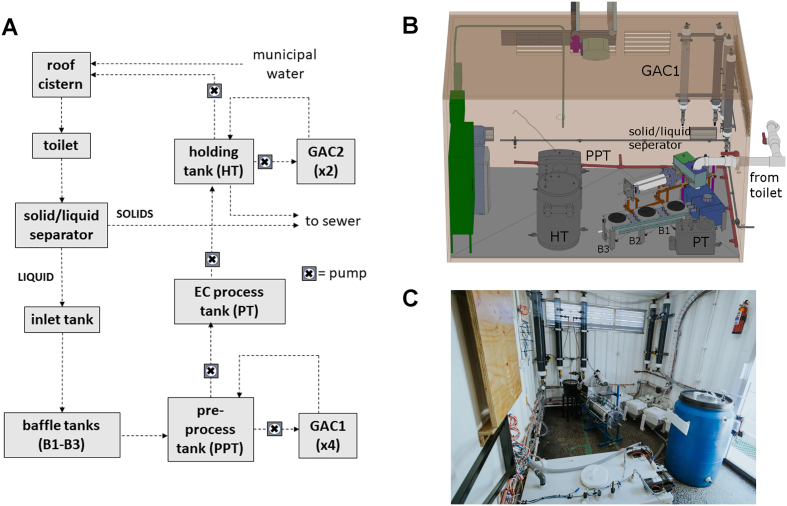
The prototype liquid treatment system. **A:** Process flow diagram of liquids and solids in the system. In these studies, the roof cistern was available to receive treated liquid and/or municipal water, separated solids and excess treated liquid were diverted to the sewer. **B:** Computer-aided design (CAD) rendering of the planned layout of the liquid treatment system in the CAB. Note that the GAC2 columns are not shown, because they are mounted on the cut-away wall in this rendering. **C:** The fully installed system in the CAB (photo credit: Jeff Hennessy.)

The prototype system tested was based on the system described previously (Rogers et al., 2018) and installed in a building adjacent to the CAB with several modifications for the Durban test site (Fig. 1). To ensure CAB services were not interrupted by system maintenance or downtime, the CAB effluent could be diverted to the municipal sewer line. The outlet from a single toilet was connected via a horizontal pipe (75 mm in diameter, 1 m long, with a 2-degree downward slope) that ran out of the CAB to an adjacent container which housed the prototype system and onsite laboratory. The outlet of the horizontal pipe was connected to a solid/liquid separator, which diverted solids to the sewer and liquids to the prototype system. The laboratory-based system was designed for 30 uses/day with a low-flush volume (1.5–2.0 L) toilet; since the toilets in the CAB used a 6 L flush, an inlet tank (75 L) was added prior to the settling tanks to buffer the increased flow of liquid with each flush, and the pre-process tank (PPT) volume was increased to 180 L to accommodate up to one day's anticipated influent volume with the same number of uses. This tank was connected to four granular activated carbon (GAC) filters operated in a parallel configuration. The post-process holding tank (HT) volume was also increased to 180 L and was connected to two GAC filters operated in parallel. Treated liquid could be discharged to the sewer or pumped to a cistern on the roof of the CAB (260 L) with an auxiliary connection to the municipal water supply that supplied the cistern flush in the test toilet.

To further investigate the suitability of this technology to regions lacking sanitation infrastructure such as India and sub-Saharan Africa, emphasis was placed on locally sourcing and manufacturing prototype components and spare parts. The system had previously been fabricated and trialed in India, so several of the system components including the control panel, tanks and solids collection unit fabricated in India were shipped to South Africa. The pumps, hardware for the GAC columns, and the electrochemical cell and mixer were shipped from the USA. The GAC media for the columns was purchased off the shelf from Rotocarb (Olifantsfontein, South Africa), a local company that specializes in activated carbon. Hardware required to fabricate an additional GAC column was sourced from a local pipe and plumbing supplies shop in South Africa to replace one column which was damaged during shipping.

### 2.2 Test site

The prototype was field tested in a well-established informal settlement in Durban with approximately 350–400 households. The settlement's council, elected by the community, manage residents and activities taking place in the settlement, including approving field testing activities. With the governance of the council, the community is stable and well established. Most people in the community are living below the upper poverty line, which is set at R1,227 per person per month (pppm) in South Africa (Statistics South Africa, 2019). The main source of water and sanitation for the community are CABs, but approximately 14% of residents use unventilated pit latrines and 4% practice open defecation. There are 6 CABs with approximately 60 households per CAB, however the CABS are closed overnight, so residents often use buckets for urination and defecation in their homes and dispose of the waste in or around the CABs the following day (C. Sutherland, University of KwaZulu-Natal, personal communication.)

A study consisting of local qualitative interviews, email surveys with experts and a focus group of people in Durban from different political, and environmental backgrounds and diverse in faith, race, gender and ethnicity showed that wastewater recycling was regarded a viable but not favorable solution in dealing with water shortages (Wilson and Pfaff, 2008). For this field study, a team of social scientists from the University of KwaZulu-Natal's (UKZN) School of Built Environment and Development Studies (SoBEDS), the Community Liaison Officer (CLO) from EWS, and project managers from Khanyisa Projects addressed the community councilors throughout each project phase. A community CLO, selected by the community leadership, served as the main point of contact onsite during installation, testing and decommissioning. The community was assured the recycling of water would be carried out only after the water quality met municipality's standards during field testing. This was important as the community regards the municipality as the institution responsible for the provision of safe sanitation facilities. In addition, information campaigns with the community in the form of workshops and instructional pamphlets posted in each toilet stall reminded users not to dispose of trash in the toilets. Throughout the field trial, minimal trash was observed flushed into the prototype system, and did not pose a challenge at this field testing site.

### 2.3 Onsite field measurements

Total energy consumption of the system was manually recorded during operation at the beginning and end of each work shift from an Onesto KM4S100C Counter Type kWh Energy Meter, installed inside of the container housing the treatment technology. Total liquid flowing into the system was manually recorded from an ASM LXHP SA1508 flow meter installed on the water line feeding the toilet cistern connected to the prototype.

Sludge volumes in the inlet and baffles were estimated by illuminating the base of the tanks and measuring the observed sludge height with a ruler. An average from four sampling points in the inlet tank was used for the approximate inlet sludge height, as sludge did not settle evenly throughout the tank. Baffles were sampled from the center of the tanks.

Water quality measurements were recorded throughout the treatment process in the inlet, third baffle (B3), pre-process, and holding tanks. Generally, daily measurements were taken after the system was opened to users for several hours. The water quality parameters measured routinely are listed in Table 1, along with the frequency with which they were tested. Tap water served as the blank for color comparison. All ammonia and free and total chlorine samples above the detection limit of the Hach DR900 were diluted using distilled water. Increased frequency of nitrogen testing was required for process monitoring during the sixth month of field testing, so simple methods for onsite analysis were selected, including Hach nitrate and nitrite test strips. On days where liquid was processed electrochemically, ammonia samples were also taken at the beginning and end of liquid processing.

**Table 1:** Testing frequency of water quality parameters. Instruments were used in accordance with the manufacturer's manual and where multiple methods are possible, the method number is stated. *Represents data collected for the second half of the trial (January to March).

Parameter	Onsite or off-site testing	Frequency	Instrument/Method
Color	Onsite	Daily	Hach Color Test Kit, Model CO-1
	Offsite	Monthly	Hach Color Test Kit, Model CO-1
Turbidity	Onsite	Daily	Hach 2100P Portable Turbidimeter
	Offsite	Monthly	Hach 2100Q Portable Turbidimeter
ORP, pH, conductivity	Onsite	Daily	Myron L Ultrameter II
	Offsite	Monthly	Hach Sension MM374
Nitrate & nitrite	Onsite	Weekly*	Hach Test Strips
	Offsite	Monthly	Merck Spectroquant Prove 300
Ammonia	Onsite	Daily*	Hach DR 900, Method 8155
Ammonium	Offsite	Weekly	Merck Spectroquant Prove 300
Free and total chlorine	Onsite	During liquid processing	Hach DR 900, Methods 8021 and 8167
Chloride	Offsite	Monthly	Merck Spectroquant Prove 300
TN	Offsite	Monthly	Merck Lovibond Spectroquant Prove 300
TP	Offsite	Monthly	Merck Spectroquant Prove 300
COD	Offsite	Weekly	Merck Spectroquant Prove 300; Hach DR900, Method 435
*E. coli*	Offsite	Monthly	Merck MC-Media Pad
Total coliforms	Offsite	Monthly	Merck MC-Media Pad

### 2.4 Off-site lab measurements

Samples were collected from the test site and sent for analysis at the Pollution Research Group (PRG) within UKZN for additional testing and system performance validation.

*E. coli*, total coliforms, and fecal coliforms were measured to estimate the level of microbial contamination in the influent and effluent. Concentrated samples were diluted with distilled water or appropriate buffer solutions using sterilized equipment. One ml of sample was dropped in the middle of a Petrifilm plate (Merck 3M™ Petrifilm™ *E. coli/*Coliform Count Plates), dispersed and incubated for 48 h at 32–35 °C. Colonies of *E. coli* and total coliforms were counted as CFU/ml.

## 3 Results and discussion

### 3.1 Toilet use patterns from flow data

The prototype system only accepted wastewater when the onsite engineering team was present, typically ~6 h/day on weekdays only. Flow data were collected over 92 testing days, during which the average liquid flow into the prototype system was 36 L. The cistern was set for a 6-L flush volume, which corresponds to an average of 6 uses of the test stall per day during the hours when the engineering team was present.

On 3–4 days, high influent rates (over 100 L in 6 h) were noted. These were correlated with toilet cistern leaks, on account of a faulty flushing mechanism, and an increase in solid material observed in the PPT, due to inadequate residence time in the baffle tanks for settling. This infiltration of dilute tap water may have affected treatment processing, as higher levels of COD, color and turbidity from unsettled solids entered the process tank and lower levels of chloride from urine were present to aid in disinfection.

### 3.2 Liquid processing performance

**Fig. 2:**
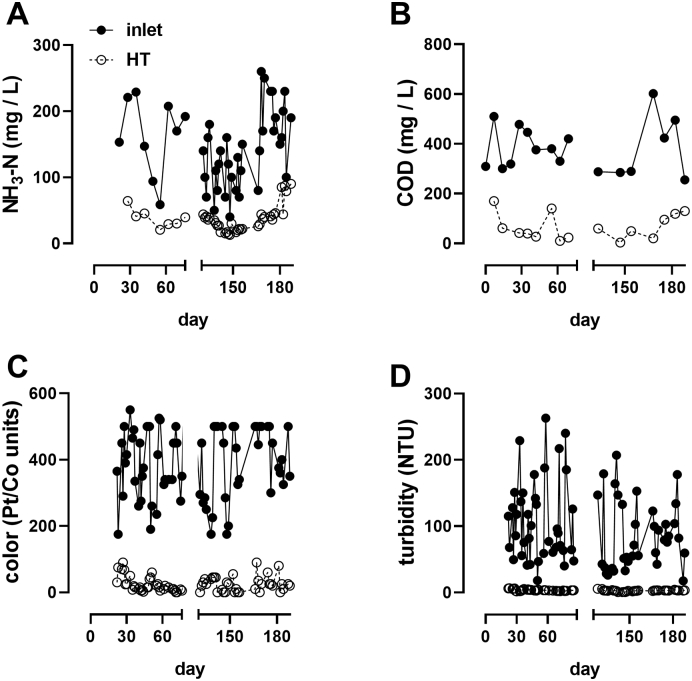
System performance over two testing periods. Data are from samples collected daily (**A**, **C**, and **D**) or weekly (**B**) from the inlet and holding tank (HT). The gap on the x-axes indicates a period of full system shutdown (32 days).

**Fig. 3:**
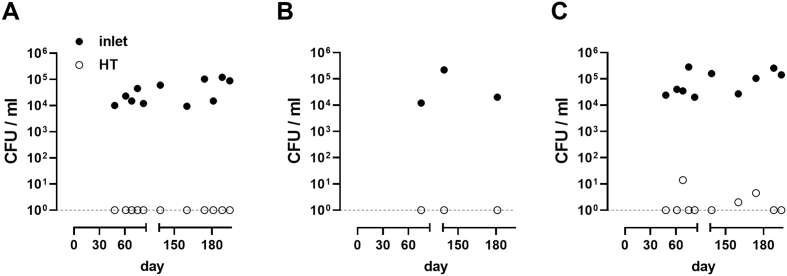
Disinfection efficacy. Shown are individual measurements for *E. coli* (**A**), fecal coliforms (**B**), and total coliforms (**C**) taken from the inlet tank and the post-process holding tank (HT) over the course of field testing. Dotted lines indicate the detection limit (1 CFU/ml).

Table 2 shows influent and effluent characteristics and removal efficiencies summarized over the entire field trial. The system was run for four months in 2018, followed by a 32-day system shutdown over December, and then run for an additional three months in 2019. System performance did not change despite the month of inactivity during system shutdown (Fig. 2), and inactivation of bacterial species to near or below detection limits was achieved consistently throughout field testing (Fig. 3).

**Table 2:** Summary of liquid system performance. Data are mean ± S.D. Effluent standards are in grey; EWS refers to local (eThekwini Water and Sanitation) standards for liquid reuse, ISO 30500 refers to standard for effluent reuse in non-sewered sanitation systems (ISO, 2018); for COD and TSS the lower numbers refer to Category A (unrestricted reuse, including toilet flushing) and the higher numbers to Category B (restricted reuse). FCl: free chlorine, TN: total nitrogen, TP: total phosphorus.

Parameter	Inlet	Outlet	% reduction (average)	n	EWS		ISO 30500
					Average	Max	
COD (mg/L)	402 ± 95	61 ± 49	85	14	50	150	≤50/150
TSS (mg/L)	63 ± 30	23 ± 13	65	8	10	30	≤10/30
Turbidity (NTU)	97 ± 57	3 ± 1	97	78	5	10	–
pH	8.0 ± 0.4	7.0 ± 0.6	–	80	6–9		6–9
Residual FCl (mg/L)	–	2.0 ± 3.5	–	36	> 0.5	–	–
TN (mg/L)	186 ± 49	102 ± 20	45	10	–	–	70% reduction
TP (mg/L)	17 ± 9	14 ± 12	20	8	–	–	80% reduction

**Fig. 4:**
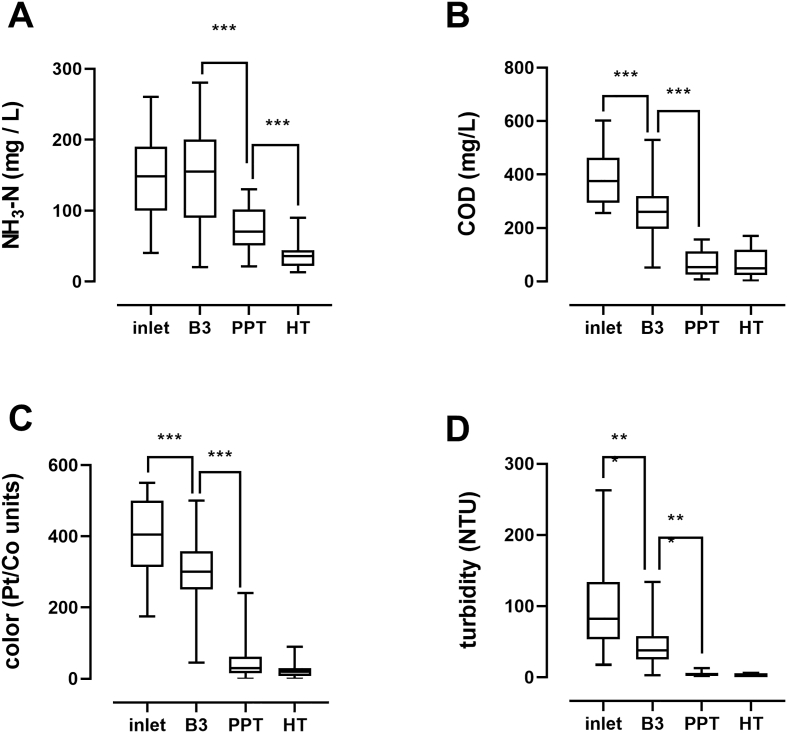
Water quality parameters at different stages of treatment. Data shown were collected throughout the field study; lines indicate medians, boxes 25th and 75th percentiles, error bars are minimum and maximum values; *n* = 44 (**A**), 17 (**B**), 76 (**C**), 75 (**D**). One-way ANOVA with a Sidak's multiple comparisons test was used to compare measurements between inlet and B3, B3 and the PPT, and the PPT and HT. *** = *p* < .001 for the comparisons indicated. Statistical analysis was performed with GraphPad Prism v. 8.0.1.

Select water quality parameters at different stages of the treatment process are shown in Fig. 4. Of these, only ammonia was not significantly reduced between the inlet and third baffle tank (B3), consistent with a significant contribution of settling to the overall reduction of COD, color, and turbidity (Hawkins et al., 2019). Ammonia, COD, color, and turbidity were all significantly reduced between B3 and the PPT, indicating the contribution of the first set of GAC columns (GAC1) to improving water quality. Interestingly, there were no significant reductions in COD, color, or turbidity between the PPT and the HT, indicating that neither the electrochemical process nor the second set of GAC columns (GAC2) significantly contributed to the overall reduction of these parameters by the system. This is consistent with previous observations in laboratory testing of an earlier prototype that the electrochemical process had no significant effect on these parameters and that GAC2 only accounted for ~2% of the overall COD removal from blackwater (Rogers et al., 2018). The difference in ammonia between the PPT (76 mg/L on average) and HT (37 mg/L on average) was significant. In a subset of processing runs (*n* = 24), ammonia concentrations were measured in the process tank at the beginning and end of the electrochemical process, with an average reduction of 18 mg/L, likely via formation of monochloramines, dichloramines, nitrite, nitrate, and/or N_2_ gas.

**Fig. 5:**
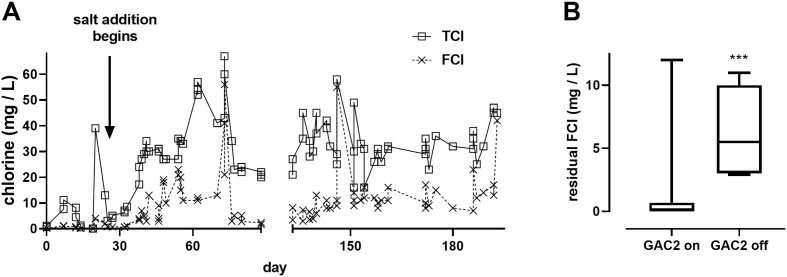
Chlorine generation and residuals. **A**: FCl and TCl measured in the process tank at the end of the electrochemical process during commissioning and testing. **B**: Residual FCl measured in the holding tank during periods when the GAC2 column was and was not online. Lines indicate medians, boxes 25th and 75th percentiles, error bars are minimum and maximum values; *n* = 26 and 10 for GAC2 on and GAC2 off, respectively. Data were compared by unpaired *t*-test, *** = *p* < .001. Statistical analysis was performed with GraphPad Prism v. 8.0.1.

Throughout the test period, chloride concentrations were too low in the influent to consistently generate enough chlorine for reliable disinfection. To remedy this, 50 g of table salt per 30 L (1.7 g/L) were added at the beginning of the electrochemical cell disinfection process to supply the chlorine required for disinfection. With salt added, free (FCl) and total chlorine (TCl) were consistently generated during the 2-hour electrochemical processing, with an average of 11 ± 10 mg/L FCl and 31 ± 12 mg/L TCl (Fig. 5A).

**Fig. 6:**
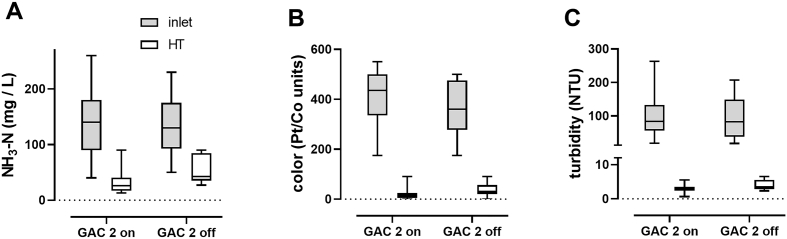
Impact of GAC2 on water quality parameters. Data shown are from samples collected at the inlet and HT during days when the GAC2 was either on or off as indicated. Lines indicate medians, boxes 25th and 75th percentiles, error bars are minimum and maximum values; *n* = 21 and 16 for GAC2 on and GAC2 off, respectively, in (A), 55 and 21 in (B), and 57 and 21 in (C). Data were compared by two-way ANOVA with a Sidak's multiple comparison test with GraphPad v 8.0.1. In all data sets the effect of GAC (on vs. off) was not significant.

Despite robust FCl generation by the electrochemical process, residual FCl levels in the HT were frequently lower than the minimum value of 0.5 mg/L indicated by local standards for water reuse to inhibit bacterial regrowth (see Table 2). To test whether GAC2 was removing too much of the residual FCl, FCl was measured in the HT with and without the GAC2 columns running. When the GAC2 columns were switched off, residual chlorine in the HT significantly increased from 0.8 ± 2.3 to 6.2 ± 3.0 mg/L FCl (Fig. 5B). Taking GAC2 offline also resulted in slight increases in ammonia, color, and turbidity in the HT, though none of these differences were statistically significant (Fig. 6). However, it is worth noting that while the increase in average turbidity was quite small (from 3 to 4 NTU) and not statistically significant, this is a potentially substantial increase when compared with the strict local standards required for reuse (5 NTU, see Table 2).

### 3.3 System energy requirements

Energy measurements from the onsite energy meter were recorded at the beginning and end of five liquid processes. These measurements were used to calculate hourly energy consumption for the liquid processing by electrolysis alone (E_E_) using the following equation:

(1) $$E_{E}=E_{\mathrm{LP}}-E_{B}-E_{C}$$

where

E_E_ = Hourly energy for electrolysis
E_LP_ = Hourly energy of total liquid processing
E_B_ = Hourly energy for the container baseline (lights, sample refrigerator, independent control panel)
E_C_ = Hourly energy for GAC1 columns operation

E_B_ and E_C_ were calculated by measuring nighttime energy consumption with and without GAC columns running. Nighttime energy consumption without GAC columns running was taken as E_B_ and the difference between nighttime readings with and without GAC1 operation were assumed to be E_C_. Total energy per liter processed (E_T_) was calculated using the following equation

(2) $$E_{T}=\left(E_{E}/V_{E}\right)*T_{E}+\left(E_{C}/V_{C}\right)*T_{C}$$

where

E_T_ = Energy requirement per L processed
V_E_ = volume of electrochemical cell process tank (30 L)
T_E_ = Duration of electrochemical treatment (2 h)
V_C_ = Maximum volume of PPT (200 L)
T_C_ = Assumed residence time for GAC1 (8 h)

Based on these assumptions, the prototype operates at 15.2 Wh/L processed (54.7 kJ/L processed). For comparison, raising the temperature of water from 20 °C to 100 °C requires 93 Wh/L at 100% efficiency; with a device working at 80% efficiency (e.g., a typical electric tea kettle) the requirement would be 116 Wh/L. GAC2 energy requirements were not included, as future prototype design will not include GAC2 based off the findings discussed in Fig. 6.

### 3.4 System maintenance

#### 3.4.1 Routine system maintenance

Regular cleaning of the GAC pump suction filters was required approximately every two months to prevent biofilm growth from disrupting water flow. In addition, the steel mixer shaft used during the electrochemical process had to be replaced after eight months of field testing due to corrosion. Regular leak checks and tightening of fittings, particularly on the GAC columns, were carried out as part of scheduled maintenance activities. Frequent cleaning of the solid/liquid separator was required due to toilet paper jamming the roller mechanism (see Section 3.5.2).

#### 3.4.2 Granular activated carbon

Backwashing of the four GAC1 columns located before the electrochemical disinfection step was required twice during the eight months of field testing. The first backwashing was performed immediately prior to the December shutdown (see Section 3.2) following 4 months of service and 1733 L of wastewater passing through the columns. This was prompted by observation of excessive liquid backing up on the top of the GAC1 columns. The second backwash event occurred following another backup 3 months after re-opening (1533 L wastewater treated). No diminished performance of the GAC1 (color and COD removal) was observed during these field tests, indicating that longer field tests will be required to determine GAC service lifetime.

#### 3.4.3 Sludge accumulation

During the eight months of field testing, removal of sludge from the settling process was not required (Fig. 5), but sludge levels were monitored. Sludge volumes were estimated from settled sludge levels in the inlet and baffle tanks prior to draining for the December shutdown and again for the overall system closure. Before the December closure (1733 L of wastewater treated) approximately 8.7 L of sludge had accumulated in the inlet tank, and approximately 1.0 L–1.9 L had accumulated in each of the baffle tanks. In all cases this represented <20% of the total tank volume. Prior to system closure (an additional 2182 L of wastewater treated), the corresponding estimated sludge volumes were 6.5 L in the inlet tank and 1.2–1.4 L in each of the baffle tanks.

### 3.5 Performance in the South African context

#### 3.5.1 Source water

As noted in section 3.2, the low levels of chloride available in the influent required 50 g of table salt to be added to each electrochemical cell process to produce enough chlorine for disinfection. Averages across 7 wastewater treatment plants under EWS jurisdiction reported 23 mg/L chloride in the effluent (eThekwini Municipality, 2019a). For comparison, a similar prototype tested in Tamil Nadu, India did not require salt addition, as source water contained an average of 330 mg/L chloride (unpublished data). In addition, low alkalinity in the influent required 30 g of sodium bicarbonate to be added to electrochemical processes when the pH dropped below 5.0. Thirteen out of 94 processes required addition of bicarbonate. Though effluent recycling as cistern flush was not tested at this field site, salt and bicarbonate addition would likely be unnecessary once steady state was achieved in the recycled process liquid (Hawkins et al., 2017).

#### 3.5.2 Solid/liquid separation

**Fig. 7:**
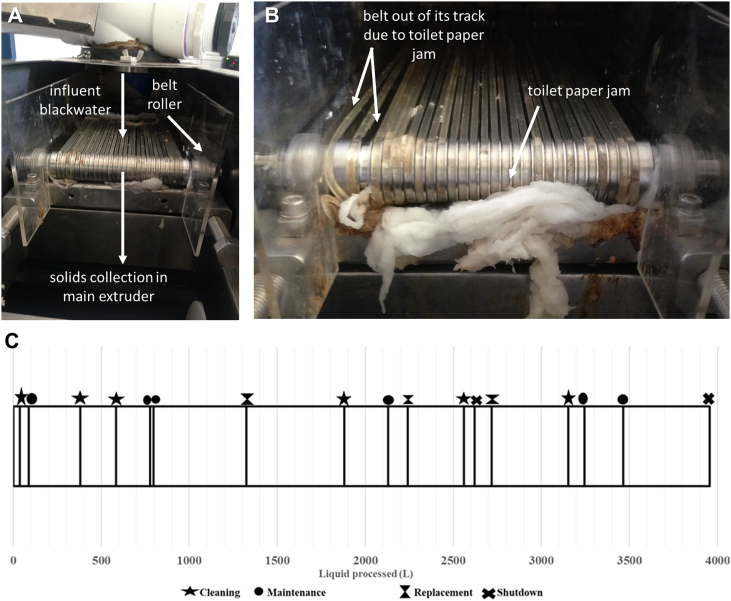
Solid/liquid separator failure. **A:** Solid/liquid separator mechanism with the direction of flow indicated. Liquids drain through the “conveyor belts” while solids are transported into the main extruder. **B:** Toilet paper jammed beneath the rotating belt resulting in the bands jumping out of their tracks and eventually breaking. **C:** Timeline showing the volume of liquid waste passed through the separation mechanism before solid/liquid separator cleaning or maintenance was performed.

The mechanism installed for solid/liquid separation simulated a conveyor belt, with the belt consisting of an array of single bands to allow the liquid waste to drain through the belt into the inlet tank while leaving solids on the belts (Fig. 7A). The belts rotated, moving solids into the main extruder, where they were disposed of via the municipal sewer. As the belt rotated, toilet paper would stick in between the bands and the motorized shaft, resulting in increased resistance on the bands (Fig. 7B). This caused some of the bands to stop rotating, which prevented solids from moving from the inlet point to the main extruder. Eventually, the increased resistance and friction resulted in the bands snapping. Occasional mechanical faults occurred due to the high resistance including a broken motor coupling. Regular maintenance of the solid/liquid separator was therefore required including scraping out toilet paper from between bands and between the belt and shaft, removing the solid/liquid separator cassette from the housing for cleaning, removing and replacing all damaged or snapped bands and repairing and replacing couplings. Two solid/liquid separator cassettes were used interchangeably so the system could continue operating while one separator was being repaired. Fig. 7C shows the volumes of liquid waste passing through each of the solid/liquid separators between cleaning, maintenance, or replacement events.

In other field tests of this system, the prototype sat directly underneath the toilet, allowing the effluent to drop directly onto the belts for solid/liquid separation. Because this much vertical space was not available at the test site in Durban, effluent from the toilet flowed approximately 1 m down a 2° angle sloped pipe onto the belt. Although the belt separator proved inadequate for handling toilet paper, these studies did demonstrate that placement of the separator directly under the toilet was not required for effective feces separation from the liquid stream. Demonstration of multiple configurations allows for smoother transitions from the field-testing to commercialization, as the spaces available for installation of these systems will vary.

### 3.6 Gaps in meeting ISO 30500 and local standards and opportunities for improvement

The ability of this technology to consistently meet relevant standards for water reuse is critical to its adoption. The recently developed ISO 30500 standard for non-sewered sanitation systems includes specific requirements for effluent quality and biological safety. In terms of effluent quality, the system effluent consistently met the category B standards for COD and TSS, which is adequate for surface discharge or other restricted urban use, but not for irrigation or toilet flushing (category A). EWS (the local water authority at this test site) has comparable standards for reuse to the ISO Category A standards for TSS and COD, and for pH (see Table 2); because the TSS and COD did not consistently meet this standard, the treated water was not recycled via the roof cistern in these studies. However, the gaps in meeting these standards are small: 11 and 13 mg/L on average for COD and TSS, respectively. It is likely that improving removal of TSS to the category A standard (e.g., by filtration) will also improve COD removal (Hawkins et al., 2018), as organic material from feces is the main contributor to TSS in blackwater streams.

pH met the standards in the majority of tests, except as noted in Section 3.5.1, indicating that some modifications to this system may also be necessary for successful deployment in areas with acidic or low-alkalinity source waters. EWS also places additional requirements on turbidity and free chlorine; turbidity standards were consistently met in these studies, but meeting the free chlorine standard was largely dependent on whether GAC2 was operational or not. Finally, significant gaps remain in meeting the ISO 30500 for TN and TP reduction; this is a challenge to non-sewered sanitation systems that do not rely on biological treatment processes generally (Trotochaud et al., 2019), and a topic of ongoing research and development.

In terms of biological safety, the influent and HT were monitored for *E. coli* which is the enteric bacterial pathogen indicated by ISO 30500. *E. coli* were below the detection limit (1 CFU/ml) in all HT samples tested; however, the standard indicates a maximum concentration of 100 CFU per L (or 0.1 CFU/ml). Thus, testing with larger sample volumes will be required to confirm that the system meets the standard. However, given that the samples were taken from the HT which has a fairly long residence time (~3–5 days depending on volume of use) and all samples came back negative (indicating no re-growth in treated effluent) it is highly likely that this standard could be met.

Although helminths were not spiked in the influent, samples were taken monthly to ensure the final product water was safe for use. No viable helminth ova were found in the final product water even on occasions when small numbers of helminth were detected in the influent, suggesting that helminths were not present in this user population. However, the limited data collected are insufficient to demonstrate the system's sustained ability to remove helminths. In order to meet ISO 30500 standards and to have confidence in the system's removal or inactivation of helminth ova, further testing would be required with a known concentration of helminth ova spiked in the influent to the system. ISO 30500 also requires demonstrated inactivation of MS2 Coliphage and *C. perfringens* spores as surrogates for enteric viruses and Protozoa, respectively. These will need to be addressed in future laboratory and field studies.

## 4 Conclusions

The system performed well over 8 months of field testing in Durban, South Africa. Effluent water quality was reliably biologically safe and met or nearly met most ISO and local standards for reuse, though effective reduction of nutrients (particularly phosphorus) remains an unmet need. A prolonged system shutdown and restart did not adversely affect performance, a highly desirable technology feature which is hard to achieve with systems relying on biological treatment. The activated carbon columns functioned without loss of performance throughout the study, requiring infrequent backwashing and no GAC replacement in the eight months of operation, suggesting that these components have a long replacement interval (~1 year or greater). However, significant improvements are required for the solid/liquid separation mechanism to operate well without burdensome maintenance in a wiping context. These findings demonstrate the feasibility of this treatment approach to application in an urban/peri-urban setting and will guide further development of onsite water recycling sanitation technologies towards implementation on a wider scale.

## Author contributions statement

Tapuwa Sahondo: Investigation, Formal Analysis, Data Curation, Writing – Original Draft, Writing – Review and Editing

Sarah Hennessy: Investigation, Formal Analysis, Data Curation, Writing – Original Draft, Writing – Review and Editing

Rebecca C. Sindall: Supervision, Project Administration, Writing – Review and Editing

Hitendra Chaudhari: Methodology, Resources

Stephanie Teleski: Methodology, Resources

Brendon J. Lynch: Methodology, Resources

Katelyn L. Sellgren: Conceptualization, Supervision, Project Administration

Brian R. Stoner: Conceptualization, Funding Acquisition, Supervision, Project Administration

Sonia Grego: Conceptualization, Supervision, Project Administration, Writing – Review and Editing

Brian T. Hawkins: Conceptualization, Formal Analysis, Visualization, Supervision, Writing – Original Draft, Writing – Review and Editing

## Declaration of competing interest

The authors declare that they have no known competing financial interests or personal relationships that could have appeared to influence the work reported in this paper.
